# Differences in cohort study data affect external validation of artificial intelligence models for predictive diagnostics of dementia - lessons for translation into clinical practice

**DOI:** 10.1007/s13167-020-00216-z

**Published:** 2020-06-22

**Authors:** Colin Birkenbihl, Mohammad Asif Emon, Henri Vrooman, Sarah Westwood, Simon Lovestone, On behalf of the AddNeuroMed Consortium, Martin Hofmann-Apitius, Holger Fröhlich, Alzheimer’s Disease Neuroimaging Initiative

**Affiliations:** 1 Department of Bioinformatics, Fraunhofer Institute for Algorithms and Scientific Computing (SCAI), Schloss Birlinghoven, 53757 Sankt Augustin, Germany; 2 Bonn-Aachen International Center for IT, Rheinische Friedrich-Wilhelms-Universität Bonn, 53115 Bonn, Germany; 3 Department of Radiology and Nuclear Medicine, Erasmus MC University Medical Center, Rotterdam, Netherlands; 4 Department of Medical Informatics, Erasmus MC University Medical Center, Rotterdam, Netherlands; 5 Department of Psychiatry, Warneford Hospital, University of Oxford, Oxford, UK; 6 UCB Biosciences GmbH, Alfred-Nobel Str. 10, 40789 Monheim am Rhein, Germany

**Keywords:** Predictive preventive personalized medicine (3 PM/PPPM), Disease risk prediction, Cohort data, Model validation, Machine learning, Disease modeling, Artificial intelligence, Individualized patient profiling, Interdisciplinary, Multiprofessional, Risk modeling, Survival analysis, Bioinformatics, Alzheimer’s disease, Neurodegeneration, Precision medicine, Cohort comparison, Health data, Medical data, Data science, Translational medicine, Digital clinic, Propensity score matching, Sampling bias, Model performance, Dementia

## Abstract

Artificial intelligence (AI) approaches pose a great opportunity for individualized, pre-symptomatic disease diagnosis which plays a key role in the context of personalized, predictive, and finally preventive medicine (PPPM). However, to translate PPPM into clinical practice, it is of utmost importance that AI-based models are carefully validated. The validation process comprises several steps, one of which is testing the model on patient-level data from an independent clinical cohort study. However, recruitment criteria can bias statistical analysis of cohort study data and impede model application beyond the training data. To evaluate whether and how data from independent clinical cohort studies differ from each other, this study systematically compares the datasets collected from two major dementia cohorts, namely, the Alzheimer’s Disease Neuroimaging Initiative (ADNI) and AddNeuroMed. The presented comparison was conducted on individual feature level and revealed significant differences among both cohorts. Such systematic deviations can potentially hamper the generalizability of results which were based on a single cohort dataset. Despite identified differences, validation of a previously published, ADNI trained model for prediction of personalized dementia risk scores on 244 AddNeuroMed subjects was successful: External validation resulted in a high prediction performance of above 80% area under receiver operator characteristic curve up to 6 years before dementia diagnosis. Propensity score matching identified a subset of patients from AddNeuroMed, which showed significantly smaller demographic differences to ADNI. For these patients, an even higher prediction performance was achieved, which demonstrates the influence systematic differences between cohorts can have on validation results. In conclusion, this study exposes challenges in external validation of AI models on cohort study data and is one of the rare cases in the neurology field in which such external validation was performed. The presented model represents a proof of concept that reliable models for personalized predictive diagnostics are feasible, which, in turn, could lead to adequate disease prevention and hereby enable the PPPM paradigm in the dementia field.

**Electronic supplementary material:**

The online version of this article (10.1007/s13167-020-00216-z) contains supplementary material, which is available to authorized users.

## Introduction

Dementia is a disease manifesting in cognitive decline of patients which ultimately leads to an inability to perform activities of daily living. Subsequently, patients are in need of full-time professional care. With an increasingly aging population, it is estimated that in 2050 there will be 1.5 billion dementia cases worldwide [1]. The economic implications are tremendous: as of now, annually $600 billion are spent on dementia care globally, surpassing the costs of cancer and heart disease, and without adequate treatment or prevention, expenses will further increase [2].

Dementia is a progressive disease that likely onsets years before cognitive symptoms arise. Treating patients who are already exhibiting cognitive symptoms shows only limited success [3, 4]. Accordingly, it has been proposed to transition to the paradigm of personalized, predictive, and preventive medicine (PPPM) in order to treat patients in pre-symptomatic dementia stages, when irreversible brain damages have not yet occurred (i.e., when patients are cognitively healthy or mild cognitive impaired, MCI, the prodromal stage of dementia) [5–8]. However, pre-symptomatic dementia diagnosis remains difficult, as reliable prognostic biomarkers have yet to be developed, and therefore, up to date, diagnosis is still mainly based on cognitive function [8].

### Artificial intelligence as a powerful instrument to implement PPPM approach

Methods from the field of artificial intelligence (AI), and more specifically machine learning, pose a great opportunity to drive the transition towards the PPPM paradigm [9]. These methods involve the use of biomedical data to build (i.e., “train”) models which are capable of addressing a plethora of problems encountered in health research: Given a suitable data, they can assist diagnosis [10], model disease progression [11], identify patient subgroups for stratification [12], analyze survival chances [13], assist disease monitoring, and support appropriate therapies and medication [14].

Often, these approaches conglomerate into one crucial aspect: they model and predict disease-relevant aspects on a personalized level and can incorporate multimodal biomedical signals as predictors. Especially these personalized predictions substantiate why AI strategies are of such relevance to the PPPM paradigm.

### Pre-symptomatic personalized dementia risk diagnosis

In the context of pre-symptomatic diagnosis, so-called AI-based disease risk models allow for predicting personalized risk years of patients, before onsetting cognitive symptoms will lead to a dementia diagnosis by a clinician. The potential of these models is an earlier identification and subsequent treatment of patients, which likely increases the chances of preventing or slowing down disease progression [15]. Several factors contributing to dementia risk are known and can be used as predictors. These contain unmodifiable patient characteristics such as biological sex, age, APOEε4 allele status, and dementia-linked single nucleotide polymorphisms (SNPs) [16–18]. Additionally, a variety of modifiable variables are known to affect dementia risk such as education, physical activity, and smoking [18]. Disease risk models can combine these predictive features to estimate the personalized dementia risk of an individual. This leads to highly multivariate models that do not only rely on single biomarkers.

### Implications of training models on cohort data: the need for validation studies

The basis for training and validation of such machine learning models are data that usually originate from a particular study (e.g., observational cohort studies). Two landmark studies in the dementia field are the Alzheimer’s Disease Neuroimaging Initiative (ADNI) [19] and AddNeuroMed [20]. ADNI is one of the worldwide largest dementia cohorts that displays an unmatched degree of deep multimodal phenotyping and longitudinal follow-up. Among others, it is funded by the National Institutes of Health (NIH) and is the most referential dementia data resource with more than 1300 citations. By sharing their complete dataset, ADNI represents a prime example in the context of open science and has enabled groundbreaking advancements in dementia research. Likewise, AddNeuroMed is up to date the largest European dementia cohort and involved participants coming from six sites all across the European Union [21]. It was the first project funded by the Innovative Medicine Initiative (IMI) and paved the way for the employed joint public-private funding scheme. Like ADNI, AddNeuroMed shares all collected patient-level data with third-party researchers.

In our earlier work [22], we have used data from ADNI to develop a machine learning model that predicts an individual patient’s risk to be diagnosed with dementia. In an internal validation, the model showed a strong performance when sequentially leaving out parts of the ADNI data from model training and using them as a test set in a nested cross-validation. However, a grand challenge in biomedicine is that clinical studies are never representative of the entire population [23], since they are inherently biased by their study design. These biases can be caused by multiple reasons, some of which are inclusion and exclusion criteria, types of collected data, or sampling and laboratory procedures. Therefore, an important question is how far an artificial intelligence model trained with data from one study can generalize (i.e., achieve sufficiently high prediction performance) to patients from another study. For this purpose, the model has to be tested on independent data. This process is called external validation. External validation is usually done retrospectively and can be understood as the first step of the long-lasting validation process [24]. The steps would comprise a prospective validation study, approval as a diagnostic tool by a regulatory agency, and finally a utility assessment, which has to carefully compare the economic costs with the achievable benefit for the patient.

To enable the paradigm shift towards AI-supported translational PPPM approaches, an adequate model validation is vital. Here, a core aspect of machine learning theory is that the training and validation data are drawn from the same underlying statistical distribution. If the training data and the validation dataset originated from two significantly different populations, validation can fail because the model is not familiar with the specific values it is presented with, even though it has successfully learned the distribution underlying the training data. Therefore, a critical question is how to quantify and decide whether a patient from an external validation study is sufficiently comparable with the original training data, given the study protocols were similar. This is an essential prerequisite for an artificial intelligence model to make reliable predictions. More broadly, any kind of statistical analysis derived from two independent studies for the same medical research question is confronted with the same issue: Only if a sufficiently similar subset of patients can be identified, statistics can be expected to be directly comparable. For example, if patients differ significantly in their age distribution in two dementia studies, their cognitive impairment scores cannot be directly compared. However, a suitable subset of patients out of both studies may be identifiable that are in the same age range and thus allow for a less biased comparison.

### State of the art: cohort comparisons and dementia risk prediction

Few evaluations of the comparability of longitudinal cohort studies in the dementia domain have been made [25, 26]. All of these works focused only on a small subset of dementia-relevant features and were based on a reduced patient subset of their investigated cohorts. In conclusion, there is an unmet need for a systematic in-depth comparison of cohorts in the dementia field.

Since the appearance of our model publication, a number of alternative machine learning algorithms for predicting dementia risk have been suggested [27–30]. Our model differentiates from those, because it is able to predict dementia risk as a function of time. Additionally, to the best of our knowledge, none of the other models were externally validated.

### Novelty beyond the state of the art

Our presented work makes two major contributions: first, we statistically analyzed the differences between two important dementia cohort studies, namely, ADNI and AddNeuroMed, in order to understand and characterize their relative sampling biases. We demonstrate that substantial differences between both studies exist in demographic, clinical, and MRI features, raising concerns regarding the generalizability of statistical analysis results and more complex modeling efforts that have solely used one of these datasets. As a second major contribution, we show that, despite the existing differences between both studies, external validation of our earlier developed dementia risk model [22] demonstrated a high prediction performance of disease diagnosis (AUC = 0.81) up to 6 years before made by a clinician. To explore the effect of systematic differences between cohorts on validation performance, we used propensity score matching (PSM) [31] to identify a subset of AddNeuroMed patients which are sufficiently similar to ADNI participants with respect to a subset of key demographic features. For those subjects, an even higher prediction performance of 88% AUC was achieved, which illustrates that systematic sampling biases can significantly influence the prediction performance of AI-based models in PPPM.

We would like to highlight that, to the best of our knowledge, our model is the only artificial intelligence-based dementia risk model that has been externally validated so far (AUC = 0.81). Hence, we see the external validation of our model as an important contribution of this work, which demonstrates that, instead of solely relying on symptomatic diagnosis, a validated PPPM approach in the dementia domain is feasible.

## Material and methods

### Clinical studies and investigated features

We selected two major dementia cohorts (i.e., ADNI and AddNeuroMed) for comparison and artificial intelligence model validation. Both studies were conducted following the Declaration of Helsinki and informed consent of participants was acquired. In order to compare the selected cohorts, and to be in a position to apply an artificial intelligence model trained on ADNI data to patients from AddNeuroMed, we first had to identify variables which were jointly available in both studies. Because demographic variables are usually well defined and clinical and MRI procedures in AddNeuroMed were aligned to ADNI protocols [20, 21], we focused on demographic, clinical, and MRI variables in our comparison. In addition, we had to ensure that brain volumes were calculated identically for both cohorts. Therefore, we reprocessed raw MRI images from ADNI and AddNeuroMed using the same pipeline and brain parcellation method (see Supplementary Material). In total, 200 variables were measured in both studies and could be compared with each other. Determined by AddNeuroMed, the longest available follow-up we could investigate spanned 84 months.

### Propensity score matching

Statistical matching or PSM is a procedure used to identify comparable patients from two cohorts. The goal is to assign patients of one cohort an individual counterpart from another dataset such that the matched pair is comparable with regard to a specified set of matching features. Classically, PSM has been used to study treatment effects outside the framework of randomized controlled trials [32], e.g., in pharma-epidemiology [33].

**Fig. 1:**
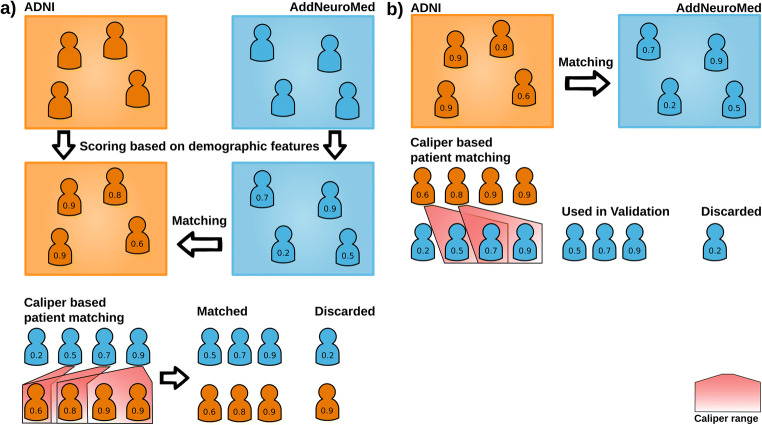
Caliper-based propensity score matching. (**A**) Procedure of caliper-based nearest neighbor propensity score matching as it was used in the comparison of ADNI and AddNeuroMed. The first step in the matching process is the calculation of a propensity score for each patient, followed by the matching of patients based on a caliper. The results are two cohorts consisting of patients similar with respect to the chosen matching features. Patients without match are discarded. (**B**) Caliper-based PSM as it was used for model validation. Only AddNeuroMed patients that found a match in ADNI were kept and used to validate the dementia risk model

Matching two dementia cohorts based on sex, age, APOEε4 status, and education level of patients will result in two sub-cohorts that are similar to each other with respect to the distribution of these matching features. PSM starts by fitting a logistic regression model which discriminates between patients of two cohorts. One class represents patients from study 1 (i.e., ADNI) and the other class study 2 (i.e., AddNeuroMed), and predictors or matching features are those clinical variables for which differences between these studies should be eliminated. The logistic regression results in a propensity score per patient in both cohorts (Fig. 1A). The score thereby represents the probability of a patient to belong to study 1. In a second step, this propensity score is then used to find suitable matching partners of ADNI patients in AddNeuroMed.

One way this can be done, which we followed here, uses the concept of a caliper [34]. For a given ADNI patient X, an AddNeuroMed patient Y is accepted as a matching partner, if their propensity score differs by at most a certain fraction of standard deviations of the propensity score. If multiple matching partners are available within the caliper range, one is selected randomly, with resampling being usually not permitted. Participants for whom no partner from the other cohort could be found within the caliper range are discarded.

The caliper can thus potentially significantly affect the matching. Althauser et al. reported that a caliper of 1 standard deviation removes approximately 75% of the initial bias, while a caliper of 0.2 can remove 98% [34]. We tested different calipers for matching: 1.5, 1.3, 1, 0.7, 0.5, 0.3, and 0.1. For each of those calipers, 100 matchings were performed and the matching quality was assessed (Supplementary Fig. 1, 2, and Supplementary Table 1). Based on this evaluation, we here decided on a caliper of 1.

To conduct PSM, we used the R package MatchIt [35]. As matching features, we selected patient age, sex, the number of full-time education years, and APOEε4 allele count. After PSM, the resulting sub-cohorts should show comparable characteristics with respect to these variables.

### Statistical cohort comparisons

We performed a comparison of ADNI and AddNeuroMed for each baseline diagnosis group separately (healthy, MCI, dementia), one before and one after PSM. We evaluated whether PSM was able to eliminate differences between ADNI and AddNeuroMed with respect to chosen matching features. Furthermore, we also investigated how PSM influenced the differences in features not matched for. To ensure robust results, we compared features for 100 matchings and set the results against those gained from comparing features in 100 randomly selected patient subgroups of the same sample size. The amount of matched/randomly selected patients from each diagnosis group can be seen in Table 1.

**Table 1:** Sample size reduction when applying PSM to ADNI and AddNeuroMed

	Healthy			MCI			Dementia		
Cohort	*n*	CC	Matched	*n*	CC	Matched	*n*	CC	Matched
ADNI	417	415	199	872	866	147	342	338	150
ANM	793	266	199	397	238	147	512	262	150

*n* number of cases before PSM, *CC* number of complete cases with regard to the matching features, *Matched* number of matched patients following the approach depicted in Fig. 1A, *MCI* mild cognitive impaired

We declared a continuous feature to be significantly different between the two cohorts if the 95% confidence interval of the difference between the population means (after correction for multiple testing via Bonferroni’s method) did not cover 0. For categorical variables (such as sex or APOEε4 status), we estimated the 95% confidence interval for the difference in proportions of each variable category (e.g., 0, 1, 2 APOEε4 risk alleles). We assessed the absolute number of significant deviations for each diagnosis cohort separately. Due to the randomness involved in the matching procedure, we repeated the comparisons 100 times, each with newly matched sub-cohorts. To evaluate if the number of found differences in matched subgroups is significantly lower than the number of differences found between random subsamples, we applied a one-tailed Wilcoxon test using an alpha level of 5%.

Since PSM cannot deal with missing data, only cases that were complete with regard to the chosen matching features were considered. After excluding incomplete cases and conducting the matching, the ADNI and AddNeuroMed sub-cohorts consisted of 199 healthy controls, 147 MCI patients, and 150 dementia cases each (Table 1 “Match”).

### Validation of an artificial intelligence-based model to predict dementia diagnosis

In our previous work [22], we proposed an artificial intelligence model based on stochastic gradient boosted decision trees (GBM) [36] for predicting the time-dependent risk of a patient to convert from a healthy or MCI state to diagnosed dementia. The model was originally trained on data from 315 cognitively normal and 609 MCI ADNI participants. Fourteen (4.4%) of the normal and 238 (39%) of the MCI patients developed dementia during the 96 months in the study. GBMs inherently perform a feature selection in the training process, which ultimately leads to sparse models. The final predictors used in the model included clinical baseline information (e.g., diagnosis, age, sex, education, and cognition scores), glucose uptake (FDG), amyloid β deposit (AV45), brain volumes (36 variables),s and genotype (APOEε4 status, 100 dementia associated SNPs, 116 polygenic pathway impact scores, and 32 principal components describing genetic variability based on 53014 SNPs within each individual). Prediction performance was assessed via 10 times repeated 10-fold cross-validation, resulting in a Harrell’s C-index of ~ 0.86. Briefly, Harrell’s C-index is a generalization of the area under the ROC curve for classification and ranges from 0 to 1, where 0.5 indicates chance level [37]. More details regarding our published model, including a comparison against several competing AI models, can be found in [22].

Since not all features used in the original model were present in AddNeuroMed, we had to restrict ourselves to the CDRSB (clinical dementia rating scale sum of boxes score) and MMSE (Mini-Mental State Examination) total scores as cognition assessments. In consequence, a revised AI model (stochastic gradient boosted decision trees—GBM) had to be trained on ADNI data. The training and subsequent evaluation procedure was identical to the one published in [22] and is described in the Supplementary Material in more detail.

In our case, the revised GBM model achieved a lower cross-validated C-index than our original one of ~ 0.83 (Supplementary Fig. 3 and Supplementary Table 2). Due to the restriction on features available in both cohorts, the revised model contained fewer features (*n* = 32) than the original one. It included 24 MRI-derived volumes of different brain regions, age, CDRSB, MMSE, baseline diagnosis (i.e., MCI or cognitively normal), 3 principal components describing genetic variance within each individual (computed from the same set of SNPs as in our original model), APOEε4 status, and 1 dementia-associated SNP (rs7364180) in the coiled-coil domain containing 134 gene (CCDC134). This revised model was subsequently evaluated on cognitively normal and MCI AddNeuroMed patients.

In addition, we investigated whether the AI model would yield better prediction performance on a subset of AddNeuroMed subjects that were more similar to ADNI patients with regard to their demographics. For that purpose, we performed PSM as shown in Fig. 1B. Based on ADNI, we scored AddNeuroMed patients and included those participants into a validation dataset who received an ADNI matching partner based on our matching variables. Additionally, baseline MMSE was included to correct for differences in cognitive impairment. No a priori stratification by baseline diagnosis was performed before PSM to avoid overoptimism. After matching, we further only included patients for whom MRI images were available. This limited the highest achievable number of validation participants to 244. The resulting average-matched validation cohort contained 164 AddNeuroMed patients of which 20 converted to dementia during the runtime of the study (Supplementary Fig. 2). To ensure that our results were robust, we repeated the validation process for 100 matchings.

## Results

### ADNI and AddNeuroMed differ significantly in key features

The presence of fundamental differences between ADNI and AddNeuroMed became evident by performing a comparison of the unmatched, full diagnosis groups. Table 2 shows an overview of the demographic characteristics of the two cohorts. With the control group as an exception, AddNeuroMed patients are on average roughly 3 years older than the ADNI population. In AddNeuroMed, the proportion of women is higher and in general there are fewer APOEε4 carriers. The most prominent difference could be observed in the education of study participants. On average, healthy ADNI participants underwent at least 4 years more education, and the cognitively affected cases showed a difference of almost 6 years compared with AddNeuroMed participants.

**Table 2:** Demographic composition of ADNI and AddNeuroMed per diagnosis

	Age	Females (%)	Education	0 *APOEε4* (%)	1 *APOEε4* (%)	2 *APOEε4* (%)
Cognitively normal controls						
ADNI	74.8	49.9	16.3	74.9	24.8	2.7
ANM	74.5	59.4	12.3	74.6	23.2	2.2
CI	[-1.33, 0.82]	**[0.02, 0.17]**	**[-4.56, -3.37]**	[-0.05, 0.09]	[-0.09, 0.05]	[-0.03, 0.02]
Mild cognitive impairment						
ADNI	73.0	40.9	15.9	49.7	39.4	10.9
ANM	76.0	54.7	10.0	60.4	35.8	3.8
CI	**[1.81, 4.25]**	**[0.06, 0.21]**	**[-6.39, -5.32]**	**[0.02, 0.19]**	[-0.12, 0.05]	**[-0.11, -0.03]**
Dementia						
ADNI	75.0	44.7	15.2	33.5	47.3	19.2
ANM	78.6	62.9	9.4	45.7	41.3	13.0
CI	**[2.17, 4.97]**	**[0.1, 0.27]**	**[-6.48, -5.1]**	**[0.03, 0.21**]	[-0.15, 0.03]	[-0.13, 0.0]

Average age and education are reported in years. *CI* multiple testing adjusted 95% confidence interval of the difference in means for education and age, and for the difference in proportions for Female and APOEε4 status. Significant intervals are emboldened. *0, 1, 2 APOEε4* fraction of individuals with 0, 1, or 2 APOEε4 alleles. *Females* proportion of female study participants. *ANM* AddNeuroMed

We could identify 200 features from the clinical, imaging, and demographic modalities that were common between ADNI and AddNeuroMed. In total, 48, 136, and 138 out of the 200 common features differed substantially between the controls, MCI, and dementia patients, respectively (Table 3 “Unmatched”). These results underline the presence of significant differences between both cohorts.

**Table 3:** Number of significant differences between ADNI and AddNeuroMed

Diagnosis	Unmatched	Random	Matched (mean, SD)	% rel. change (mean)	*p* value	Min	Max
Controls	48	26 (10.4)	22 (6.8)	-15	0.001	11	40
Mild cognitive impaired	136	67 (22.4)	23 (10.0)	-67	< 0.001	4	47
Dementia	138	66 (22.4)	17 (4.3)	-66	< 0.001	8	30

*Unmatched* number of features found significant by comparing the complete unmatched diagnosis groups. *Random* number of features found significant by comparing random subsamples with the same sample size as the matched subgroups. *Matched* mean number of significant differences found across all 100 matching and comparison runs. Standard deviation in brackets. *% rel*. *change* relative change in the number of significant features with and without PSM. *Min* minimal number of significant differences found in a single run. *p value* *p* value indicating if the amount of significant differences in matched subgroups is significantly lower compared with the random sample. *Max* maximal number of significant differences found in a single run

### Propensity score matching allows for identifying comparable subjects

**Fig. 2:**
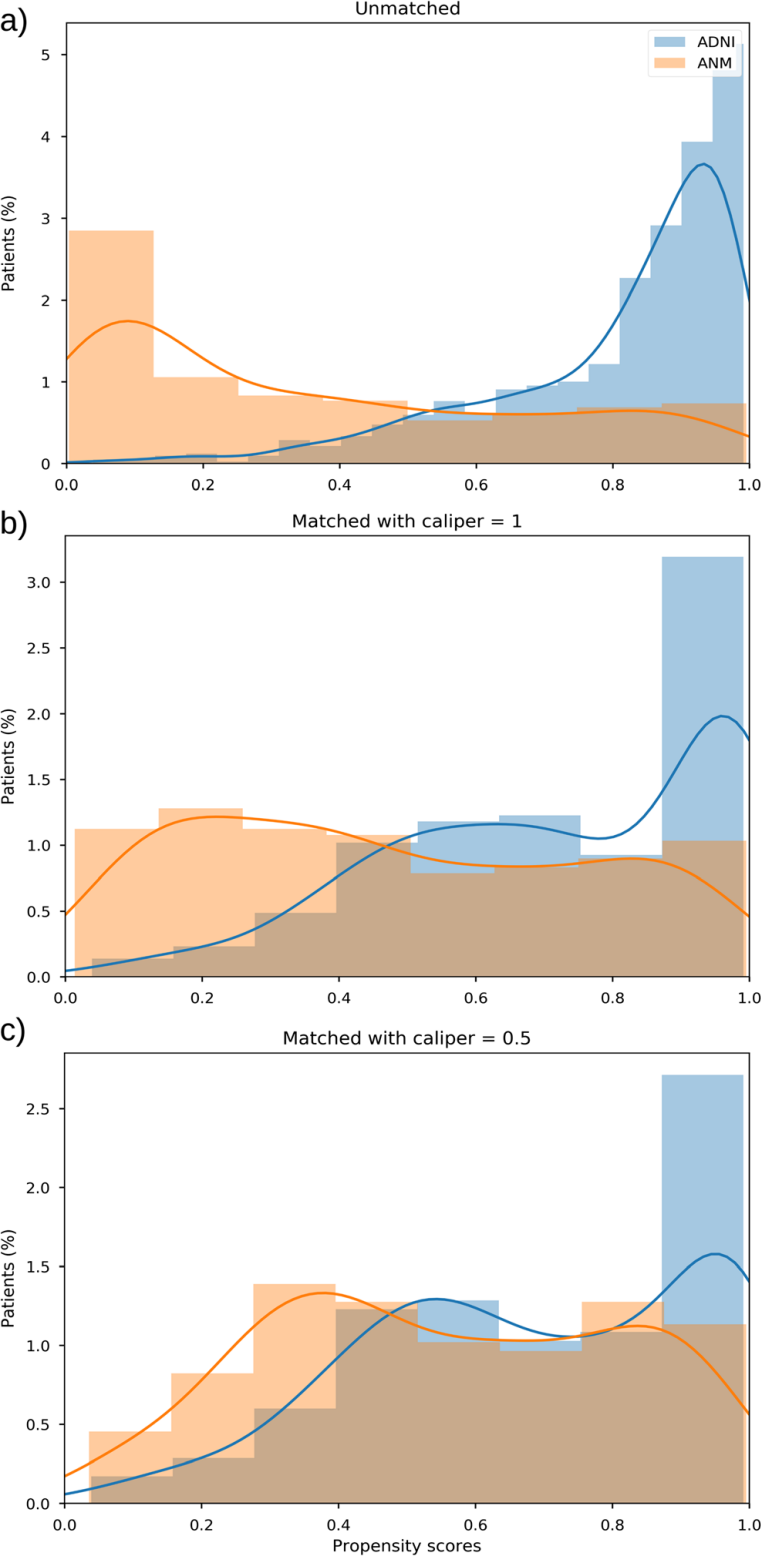
Distribution of propensity scores before and after PSM. (**A**) Scores for the full unmatched cohorts. (**B**) Scores for matched patients using a caliper of 1. (**C**) Scores for matched patients using a caliper of 0.5. *ANM* AddNeuroMed

PSM resulted in ~ 363 patients from AddNeuroMed that could principally be matched to ADNI following the PSM protocol in Fig. 1B. Keeping only patients for which MRI data was available led to a dataset comprising on average 164 patients. In Fig. 2, we show the distribution of propensity scores before and after PSM. The shift to more similar distributions after PSM highlights that differences in age, sex, MMSE, education, and APOEε4 status between matched patients from both studies are evidently reduced. Evaluation of individual confidence intervals of those features showed similar results, since significant differences observed in the matching features before PSM vanished after (Supplementary Fig. 4), the education of participants being the only exception. Hence, PSM allows for identifying more comparable subjects from AddNeuroMed with respect to key features.

Additionally, we explored whether PSM would reduce the number of significantly different features that were not used as matching variables. This was done by running 100 PSMs, each selecting the amount of matched patients previously shown in Table 1. We then compared the selected subsamples of ADNI and AddNeuroMed to identify significant differences in non-matching variables. This was done (a) in the matched subsamples and (b) as a control in 100 randomly selected patient subsets from both cohorts, which included the same number of patients as selected by PSM.

We found that the number of significantly different variables was, on average, reduced to 22 (± 7 std. dev.; i.e., reduction by 15%), 23 (± 10 std. dev.; i.e., reduction by 67%), and 17 (± 4 std. dev.; i.e., reduction by 66%) for controls, MCI, and dementia patients compared with the random samples (Table 3). Comparing the number of significant differences found in random samples and matched samples using a Wilcoxon test showed that the reduction was significant in all cases (healthy, *p* = 0.001; dementia and MCI, *p* < 0.001). This finding can be explained by the fact that matching variables are correlated with further variables.

Supplementary Fig. 4 shows which features differed consistently between AddNeuroMed and ADNI.

### Artificial intelligence model shows high prediction performance in external validation

**Fig. 3:**
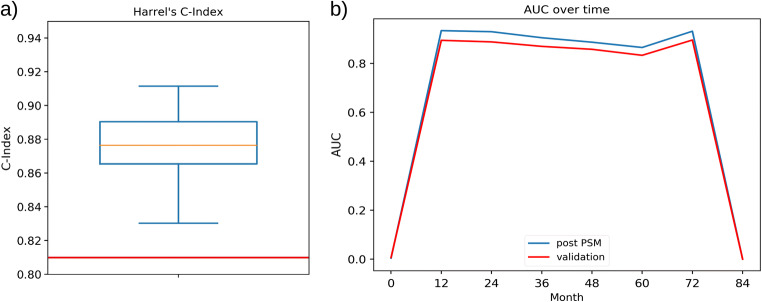
Performance of the dementia risk model on external validation and matched AddNeuroMed data calculated for 100 different matchings. (**A**) Harrell’s C-index of the model. The red line is indicates the model performance on the full unmatched AddNeuroMed cohort. (**B**) Area under the ROC over time (AUC(t)) showing the predictive performance over time before diagnosis. The standard error is plotted around the mean trajectory

We initially applied our artificial intelligence-based dementia risk model to all cognitively normal and mild cognitively impaired AddNeuroMed participants with available MRI data (*n* = 244, 30 (12%) received the diagnosis “Alzheimer’s disease” during the course of the study). Due to the highlighted differences between ADNI (our training cohort) and AddNeuroMed (our validation set), prediction performance of the model dropped from 0.83 C-index in ADNI to 0.81 C-index in AddNeuroMed (Fig. 3A). In Fig. 3B, we present the prediction performance as the area under receiver operator characteristic curve over time (AUC(t)) to show that our algorithm can predict dementia diagnosis up to 6 years prior to diagnosis with an AUC of ~ 0.8. The observed low prediction performance at month 0 is an artifact, because no conversions can take place at baseline. Likewise, after 6 years, prediction performance drops, because only few observations were available.

For comparison and motivated by the findings in the last section, we next investigated the prediction performance for AddNeuroMed subjects that were putatively similar to ADNI according to PSM. Our model made a prediction for each of the matched AddNeuroMed patients, and we repeated this procedure for 100 different matchings and averaged the performance. This resulted in a significantly higher C-Index of ~ 0.88, which is comparable with the result reported in our earlier publication using cross-validation (Fig. 3A). Similarly, the AUC at 6 years prior to diagnosis increased to ~ 0.88 as well (Fig. 3B). In conclusion, PSM successfully eradicated differences between cohorts by identifying AddNeuroMed subjects that were more similar to those in ADNI.

## Discussion

In order to take dementia treatment to the era of PPPM, pre-symptomatic diagnosis is vital. AI and machine learning methods trained on clinical cohort study data can build a foundation to enable this translation, because they can work with the highly multifactorial nature of dementia and succeed, where single biomarkers are not able to provide a reliably prediction. However, translation of AI models into clinical practice requires a sufficient multi-step validation: (i) an internal validation on the discovery cohort (done in our previous work); (ii) an external validation on a further cohort (done here); (iii) a validation via a prospective clinical study; (iv) an assessment as a diagnostic tool by a regulatory agency; and (v) a careful utility analysis, which includes health economic considerations.

### Cohort differences affect model generalizability; predictive dementia diagnosis is possible

This work demonstrated the presence of substantial differences between ADNI and AddNeuroMed, two major dementia cohort studies. Nonetheless, we were able to externally validate our model for personalized dementia risk prediction on the complete AddNeuroMed data, achieving an AUC of ~ 0.81 to predict dementia diagnosis 6 years before diagnosed by a clinician. Due to the identified differences, it is not surprising to observe a lower performance compared with the ~ 0.86 AUC we previously reported on ADNI [22]. Notably, with the help of PSM, we were able to identify a subset of AddNeuroMed subjects that were more comparable with those in ADNI with regard to demographic features. For these matched patients, a significantly higher prediction performance of ~ 0.88 AUC was observed. This again highlights the influence which systematic biases across cohorts can potentially have on the performance of AI-based approaches. We would like to emphasize that this work is one of the very rare cases in the neurology field, in which an AI model was properly validated based on a separate study. As pointed out above, such external validation is crucial to enable a paradigm shift towards an AI-based PPPM paradigm.

In general, the observable differences between ADNI and AddNeuroMed question the generalizability of published statistical analyses that in the past have only used a single dataset. Our concerns are further supported by results of Whitwell et al. [26] as well as Ferreira et al. [25], who also reported significant differences between dementia cohorts. Because there is such a strong bias in cohort data from dementia patients, from our point of view, it is extremely important that scientific findings are tested in independent cohort studies.

### Limitations and outlook

For this work, there was only a relatively small number of initially cognitively normal and MCI patients in AddNeuroMed, which later on received the dementia diagnosis (30 out of 244). Hence, additional cohort studies should be employed to further validate the presented AI model. Since each of these studies will have their own biases compared with ADNI as well, such a validation would additionally strengthen the confidence into the model. The next step in order to allow for an implementation of the presented model into a clinical context would be a dedicated prospective study.

### Expert recommendations: AI-supported personalized treatments

The crucial role that AI models can play in the process of shifting the diagnosis and treatment of dementia towards the PPPM paradigm stems mainly from their capability of performing personalized predictions. By incorporating patient-specific multivariate information, they provide disease risk assessments for individuals which can potentially impact the time as well as the type of treatment that patients receive. Thereby, reliable AI models can constitute personalized treatment algorithms that open opportunities for critical medical interventions which delay the progression of diseases or even to prevent disease onset at all. Furthermore, AI methods could even suggest the appropriate personalized treatment given the patient specific biomarker signatures. The accompanied reduction in economic costs as well as emotional burden suffered by patients and caregivers would be significant.

### Conclusion

Altogether, our work highlighted the inevitable necessity to validate AI models on separate cohort datasets to, at some point, make the translation of AI-based PPPM approaches into clinical routine [6, 24, 38]. Moreover, our work showed the non-trivial challenges that are associated with conducting such efforts. Additional real-world evidence data from clinical practice (e.g., electronic health care records) are now starting to play an increasing role in this context and could potentially help to reduce the cohort selection biases outlined here.

## Electronic supplementary material

ESM 1 (DOCX 587 kb)

## Data Availability

All used datasets are findable in official repositories and are cited accordingly. ADNI data are disseminated by the Laboratory for Neuro Imaging at the University of Southern California.

## Abbreviations

ADNI: Alzheimer’s Disease Neuroimaging Initiative
AI: artificial intelligence
AUC: area under receiver operator characteristic curve
CDRSB: clinical dementia rating sum of boxes
MCI: mild cognitive impairment
MMSE: Mini-Mental State Examination
PPPM: predictive preventive personalized medicine
PSM: propensity score matching
SNP: single nucleotide polymorphism
