# Supplementary material for: Differences in cohort study data affect external validation of artificial intelligence models for predictive diagnostics of dementia - lessons for translation into clinical practice
Source: EPMA J. 2020 Jun 22;11(3):367–76. doi: 10.1007/s13167-020-00216-z (PMC7429672; doi:10.1007/s13167-020-00216-z)
Supplement: Supplementary file 1 — (DOCX 587 kb) [file 13167_2020_216_MOESM1_ESM.docx]

**Supplementary Material: How differences in cohort study data affect external validation of artificial intelligence models for diagnosis prediction of dementia**

**Colin Birkenbihl, Mohammad Asif Emon, Henri Vrooman, Sarah Westwood, Simon Lovestone** **on behalf of the AddNeuroMed Consortium, Martin Hofmann-Apitius, Holger Fröhlich, and the Alzheimer's Disease Neuroimaging Initiative**

## Details about Propensity Score Matching

To evaluate the matchings, we fitted logistic regression models separately per baseline diagnosis using the demographic matching features as predictors. This classified the patients into either being AddNeuroMed or ADNI participants and the area under the ROC curve (AUC) was calculated. A lower AUC indicates a worse discrimination of subjects from both studies. After PSM a notable lower classification performance was observed, again indicating that patient cohorts became more comparable with regard to matching features.

**S. Table 1:** Discrimination performance before and after PSM

|  | **pre PSM** | **post PSM (SD)** |
| --- | --- | --- |
| CTL | 0.85 | 0.69 (0.01) |
| MCI | 0.87 | 0.61 (0.01) |
| Dementia | 0.89 | 0.69 (0.01) |

Average area under the ROC curve (AUC) for logistic regression classifier that try to discriminate between ADNI and AddNeuroMed patients based on the chosen matching features before and after PSM. 100 PSM matchings were considered to calculate the AUC after PSM.

###


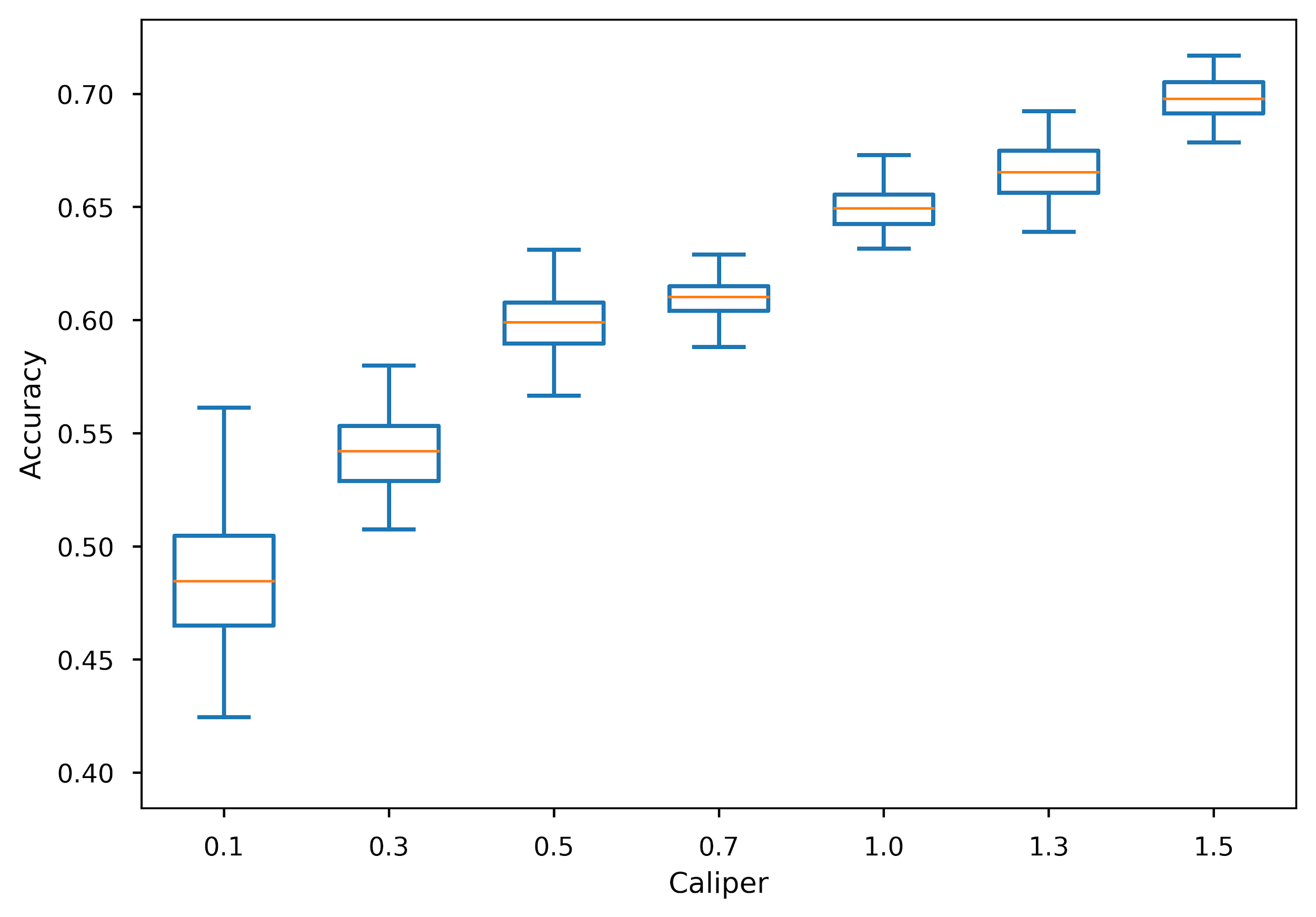


**Supplementary Figure 1:** **Influence of caliper on cohort similarity in propensity score matching**. Accuracy of a logistic regression model that discriminates between ADNI and AddNeuroMed patients based on the matching features after PSM for different caliper settings. PSM was performed as described for model validation (Fig. 1B). Note that there is an equal number of ADNI and AddNeuroMed patients after matching.


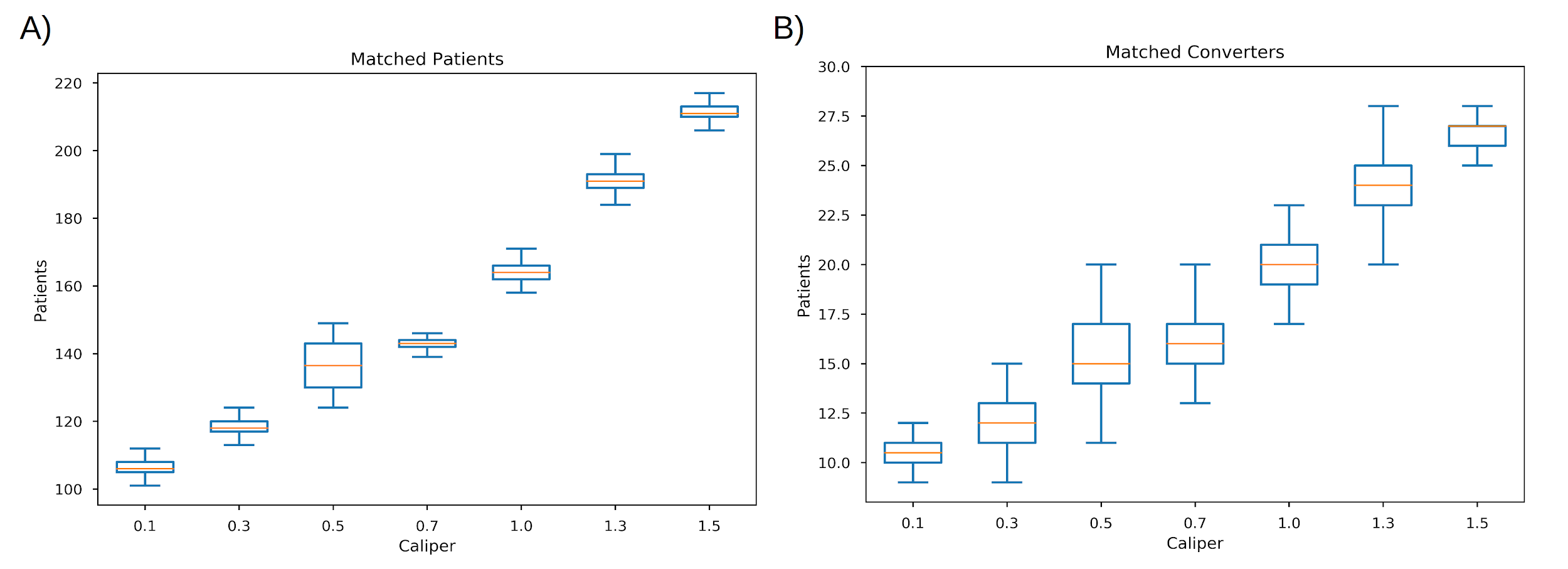
**S. Figure 2: Influence of caliper on number of patients matched.** The effect of different caliper ranges onto the number of matched AddNeuroMed participants following the PSM protocol as shown in Fig. 1B. 100 matching runs have been performed. **A)** Number of matched participants **B)** Number of matched converters.

## Volumetric Measurements for Cortical, Subcortical and Global Brain Regions

All available MR scans (T1-weighted scans) from ADNI and AddNeuroMed were quantified by an open-source, automated segmentation pipeline at the Erasmus University Medical Center, The Netherlands. The number of slices of the T1w scans varied from 160 to 196 and the in-plane resolution was 256 x 256 on average, yielding an overall voxel-size of 1.2 x 1.0 x 1.0 mm. From all available baseline scans that were present in the ADNI and AddNeuroMed cohorts, the volumes of 7 bilateral subcortical brain regions, 75 bilateral cortical regions, and 20 global brain regions or structures were calculated using a model- and surface-based automated image segmentation procedure, incorporated in the FreeSurfer Package (v.6.0, <http://surfer.nmr.mgh.harvard.edu/>). In total, the volumes of 184 brain regions were calculated using this FreeSurfer tool. Segmentation in Freesurfer was performed by rigid-body registration and nonlinear normalization of the images to a probabilistic brain atlas. In the final segmentation process, each voxel of the T1-weighted MRI volumes was fully automatically labeled as the brain region. The 75 bilateral cortical regions were based on the Destrieux cortex parcellation atlas [1]. The segmentation of subcortical regions was first described by Fischl *et al.* [2].

1. Destrieux, C., Fischl, B., Dale, A., Halgren, E. (2010). Automatic parcellation of human cortical gyri and sulci using standard anatomical nomenclature. Neuroimage, 53(1), 1-15.
2. Fischl, B., Salat, D. H., Busa, E., Albert, M., Dieterich, M., Haselgrove, C., et al. (2002). Whole brain segmentation: automated labeling of neuroanatomical structures in the human brain. Neuron, 33(3), 341-355.

## Details about Revised AI Model Training and Evaluation

Briefly, a GBM constitutes a weighted ensemble of weak decision tree classifiers (base learners) with restricted maximal depth (here 3). A higher maximal tree depth results into more complex base learners which capture higher order interactions between variables (here: up to 3-way interactions). On the other hand, a tree depth of 1 corresponds to simple decision stumps and can require longer boosting, depending on the overall optimal complexity of the GBM model. The reason is that the actual number of trees in the ensemble (and thus overall complexity of the GBM model) critically depends on the number of boosting steps, which is a tunable hyper-parameter. Depending on the maximal depth and number of decision trees, GBMs do not necessarily employ all existing features in the data, but possibly only a subset. We found the optimal hyper parameter (number of boosting steps) via an inner 10-fold cross-validation. Importantly, this was done within the outer 10 times repeated 10-fold cross-validation procedure used to evaluate prediction performance. GBM’s can deal with censored time-to-event data, as present in our application (here: time to dementia diagnosis): We want to predict the time until dementia is diagnosed. For some dementia converters such a diagnosis will be observed within the study time. However, there are also participants for which the diagnosis cannot be established during study runtime, but eventually after it ended. Their observed times-to-event are thus considered right censored. Our employed GBM implementation (R-package gbm) allows for dealing with time-to-event data by using the negative partial log-likelihood of the Cox proportional hazards model as a loss function. As typical in clinical studies ADNI data contains missing values. GBM rely on a surrogate split approach for this purpose. GBM allow for a ranking of variables according to their relevance for the model. This is done by recording the relative reduction of the loss function as a measure of variable importance. Accordingly, features with zero importance can be filtered out. Hence, GBM can be used for feature selection.


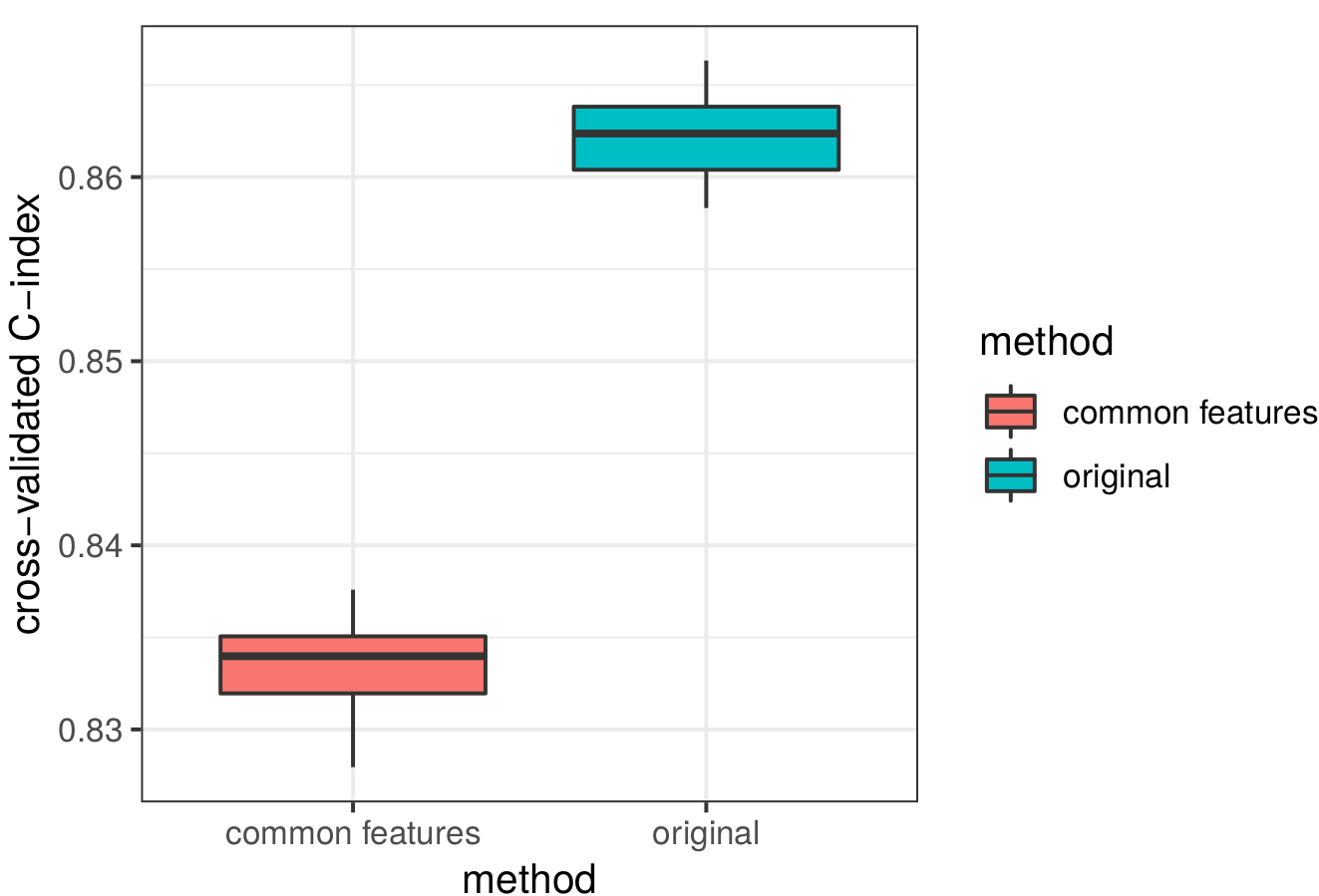


**S. Figure 3: Original model performance compared to common feature model.** Estimated prediction performance in terms of C-index (10 times repeated 10-fold cross-validation) of our original machine learning model (Khanna *et al.*, 2018) vs the revised one that only uses features that are commonly available in AddNeuroMed (red). Both machine learning models were trained with the same set of patients.


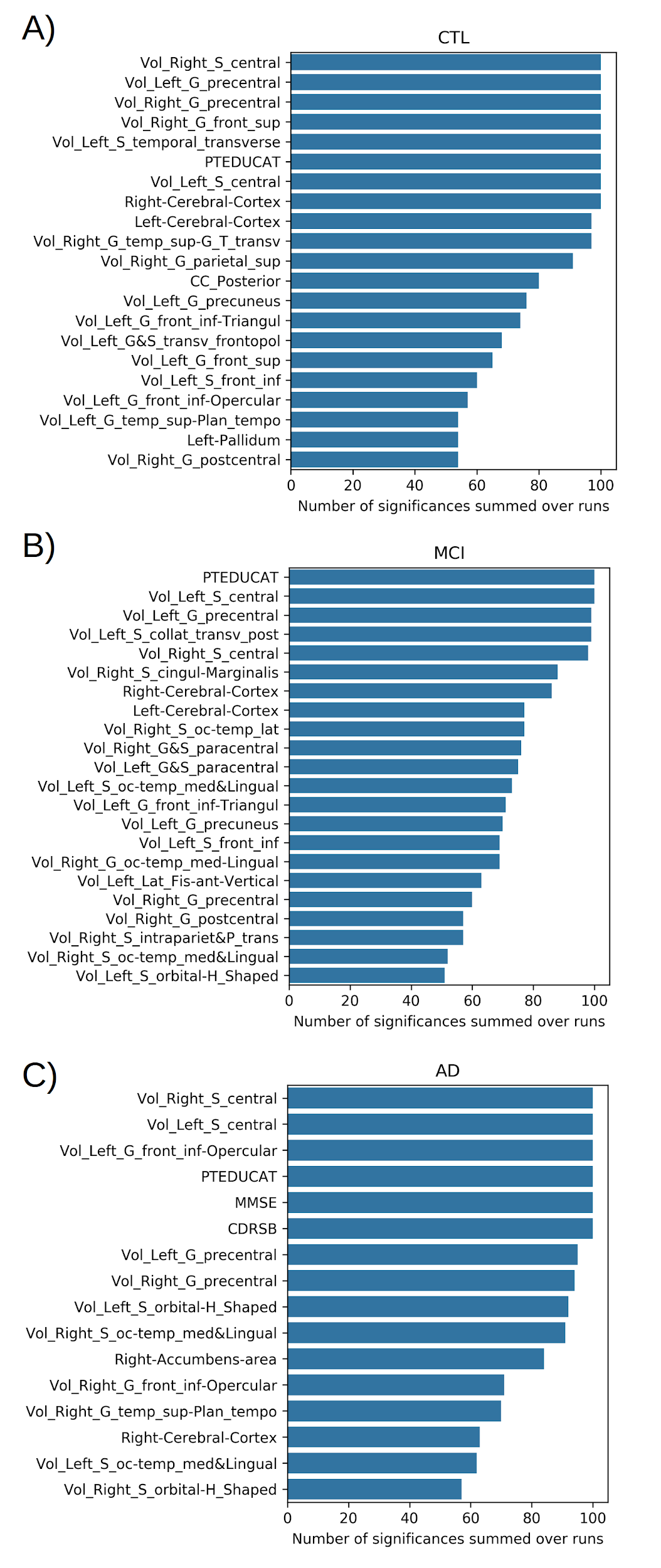


**S. Figure 4: Significantly different features between ADNI and AddNeuroMed.** Features that were found significantly different between ADNI and AddNeuroMed in at least 50 out of the 100 matching and comparison runs. Cortical MRI variables are named following the Destrieux parcellation atlas [29], one of the two cortical parcellation schemes present in FreeSurfer v6.0. **A)** Controls. **B)** MCI. **C)** AD = Dementia.

**S. Table 2:** Relative importance of predictors in revised AI model

| **Variable** | **Rel. Importance (%)** | **Missing values (%) in ADNI** | **Missing values (%) in AddNeuroMed** |
| --- | --- | --- | --- |
| Baseline diagnosis (MCI or cognitively normal) | 27.46 | 0 | 0 |
| CDRSB | 12.29 | 0 | 0.8 |
| Vol. Left.Hippocampus | 7.9 | 1.2 | 0 |
| Vol. Left Amygdala | 5.64 | 1.2 | 0 |
| Vol. Right Inf. Lat. Vent | 3.4 | 1.2 | 0 |
| Optic Chiasm | 2.89 | 1.2 | 0 |
| Vol Left G.S. subcentral | 2.73 | 1.2 | 0 |
| Vol. Brain Stem | 2.66 | 1.2 | 0 |
| MMSE | 2.52 | 0 | 0 |
| Vol. Left Inf Lat Vent | 2.52 | 1.2 | 0 |
| Vol. Right Hippocampus | 2.49 | 1.2 | 0 |
| Vol. Right G pariet inf Angular | 2.39 | 1.2 | 0 |
| EV2 | 2.21 | 0 | 18.0 |
| APOE4 | 2.2 | 0 | 3.3 |
| EV31 | 2.16 | 0 | 18.0 |
| Vol. Left G. oc. temp lat. fusifor | 1.88 | 1.2 | 0 |
| Vol. Left G. cingul Post dorsal | 1.81 | 1.2 | 0 |
| EV13 | 1.64 | 0 | 18.0 |
| Vol. Right S. circular insula inf. | 1.55 | 1.2 | 0 |
| Vol. Right Cerebral White Matter | 1.4 | 1.2 | 0 |
| AGE | 1.31 | 0 | 0 |
| Vol. Left S. intraparie P. trans. | 1.3 | 1.2 | 0 |
| Vol. Right G. S. occipital inf. | 1.01 | 1.2 | 0 |
| Vol. Left Cerebral Cortex | 0.87 | 1.2 | 0 |
| Intracranial volume | 0.83 | 1.2 | 0 |
| Vol. Right G. precuneus | 0.82 | 1.2 | 0 |
| Vol. Left Pole occipital | 0.82 | 1.2 | 0 |
| Vol. Left Thalamus Proper | 0.71 | 1.2 | 0 |
| rs7364180 | 0.7 | 0 | 18.0 |
| Vol Right S. circular insula ant | 0.66 | 1.2 | 0 |
| Vol Right S. oc. middle Lunatus | 0.64 | 1.2 | 0 |
| Vol. Left S. temporal sup. | 0.6 | 1.2 | 0 |

Features in revised machine learning model together with their relative importance values according to Friedman (2002). **EV***: Principal components calculated out of the same set of 53,014 SNPs that we used in our earlier publication (Khanna *et al.*, 2018). **Missingness:** Percentage of patients with missing data for the respective variable. During AI model training these were modeled via the surrogate variable split method that is part of the stochastic gradient boosted decision tree learning algorithm (Friedman, 2002).
